# Recent Molecular Assessment of *Plasmodium vivax* and *Plasmodium falciparum* Asymptomatic Infections in Botswana

**DOI:** 10.4269/ajtmh.21-0083

**Published:** 2021-05-03

**Authors:** Thato Motshoge, Daniel H. Haiyambo, Ruth Ayanful-Torgby, Larysa Aleksenko, Davies Ntebela, Benoit Malleret, Laurent Rénia, Elias Peloewetse, Giacomo Maria Paganotti, Isaac K. Quaye

**Affiliations:** 1 University of Botswana, Department of Biological Science, Gaborone, Botswana;; 2 University of Namibia School of Medicine, Windhoek, Namibia;; 3 Regent University College of Science and Technology, Department of Engineering, Computing and Allied Health Sciences, Accra, Ghana;; 4 Biomedical and Public Health Research Unit, Council for Scientific and Industrial Research–Water Research Institute, Council Close, Accra, Ghana;; 5 University of Lund, Department Clinical Sciences, Lund, Sweden;; 6 National Malaria Program Ministry of Health and Wellness, Gaborone, Botswana;; 7 Department of Microbiology and Immunology, Immunology Translational Research Program, Yong Loo Lin School of Medicine, Immunology Program, Life Sciences Institute, National University of Singapore, Singapore;; 8 Singapore Immunology Network (SIgN), Agency for Science, Technology and Research (A*STAR), Biopolis, Singapore;; 9 A*STAR Infectious Diseases Laboratories, Agency for Science, Technology and Research (A*STAR), Biopolis, Singapore;; 10 Botswana–University of Pennsylvania Partnership, University of Botswana, Gaborone, Botswana;; 11 Division of Infectious Diseases, Perelman School of Medicine, University of Pennsylvania, Philadelphia, Pennsylvania;; 12 Department of Biomedical Sciences, Faculty of Medicine, University of Botswana, Gaborone, Botswana

## Abstract

In 2016, we reported the presence of *Plasmodium vivax* in Botswana through active case detection. A real-time PCR was used during a similar study in 10 districts to assess changes in the *P. vivax* prevalence. We assessed 1,614 children (2–13 years of age) for hemoglobin (Hb; g/dL) and *Plasmodium* parasites. The median age of all participants was 5.0 years (25^th^ percentile, 3 years; 75^th^ percentile, 8 years). The median Hb (g/dL) level was 12.1, but 18.3% of the participants had anemia (Hb < 11.0 g/dL); these participants were clustered in the younger than 5 years age group in all districts (*P* < 0.001). The risk of anemia decreased with age 5 years or older (odds ratio [OR], 0.26; 95% confidence interval [CI], 0.197–0.34; *P* < 0.001). The prevalence rates of *Plasmodium* parasites were as follows: *P. vivax*, 12.7%; *P. falciparum*, 12.7%; *P. malariae*, 0.74%; and *P. ovale* (*P. ovale curtisi*), 0.68%. Mixed infection rates were as follows: *P. falciparum* and *P. vivax*, 2.35%; *P. falciparum* and *P. ovale curtisi*, 0.56%; *P. vivax* and *P. malariae*, 0.06%; and *P. falciparum* and *P. malariae*, 0.68%. The infections were largely asymptomatic (99.6%). Using logistic regression, the risk of infection with *P. vivax* was highest in Kweneng East (OR, 6.2; 95% CI, 2.9–13.1), followed by South East (OR, 5.6; 95% CI, 2.5–12.3) and Ngami (OR, 5.1; 95% CI, 2.2–12.0). Compared to the risk of infection for children younger than 5 years, the risk of infection decreased for children 5 years or older in regions with high rates of *P. vivax and P. falciparum* infections. *P. vivax* and *P. falciparum* have expanded within the asymptomatic population in Botswana; therefore, careful attention is required for their elimination.

## INTRODUCTION

Botswana is a member of the Southern African Development Community (SADC) Elimination 8 (E8) countries that aimed to eliminate malaria by 2018.^1^ Unfortunately, the elimination target could not be achieved because of persistent factors, including the sole focus of the National Malaria Program (NMP) on *Plasmodium falciparum* (*P. falciparum*) for elimination, limited human resource capacity, and structural/logistic impediments that affect disease surveillance.

In 2016, the first report of the presence of asymptomatic *P. vivax* infections in the country was made in a collaborative venture with the NMP.^2^ Between 2015 and 2017, a similar collaboration to perform active case detection of all *Plasmodium* species that infect humans (*P. falciparum*, *P. vivax*, *P. ovale*) and *P. malariae* in schools and hospitals located in 10 districts across the country was performed to assist the NMP with the stratified data required for an effective malaria elimination plan.^3–5^ In 2019, the World Health Organization (WHO) noted that the elimination agenda in Botswana was not working and needed to be reviewed with a new approach.^6^ This was a necessary approach to align with the overall global target of malaria eradication.^7^

It has been well-established that as the rates of malaria attributable to *P. falciparum* decrease, the rates of nonfalciparum (*P. vivax*, *P. ovale*, and *P. malariae*) malaria increase.^8,9^ In addition, *P. vivax* is now well-recognized in Africa, including in Duffy-negative individuals.^10^ Both *P. malariae* and *P. ovale* are commonly found in Africa. However, unlike the two sympatric species of *P. ovale* (*P. ovale wallikeri* and *P. ovale curtisi*) that have hypnozoites stages similarly to *P. vivax*, it is unknown if *P. malariae* have hypnozoites even though they have long latency periods and can sustain infections through relapses or recrudescences.^11,12^ *P. malariae* is known to have an enhanced capability to sustained infection in humans.^13^ Several reports have shown that asymptomatic infections with all *Plasmodium* species comprise a significant portion of the burden of disease.^11,14,15^ Therefore, it is reasonable to surmise that a more effective approach to malaria elimination and eradication in Africa should consider a strategy that targets all *Plasmodium* species in parallel in asymptomatic and symptomatic infections based on accurate active case detection using highly sensitive molecular tools.^16^ It is essential for the scientific community to work closely with National Malaria Control Programs (NMCPs) to achieve this goal. By obtaining data regarding all *Plasmodium* species in Africa, the impetus for guidance regarding the elimination and eradication strategies will allow NMCPs to retool their resources appropriately.

There are geospatial limits to the transmission dynamics of *Plasmodium* species that can be missed with passive data collection, which is unable to detect submicroscopic and asymptomatic infections and how they contribute to transmission.^17,18^ The Botswana NMP is retooling to forge a collaboration to create elimination strategies and improve disease surveillance, monitoring, and evaluation.^19^ The collaboration will improve the human resources and technical capacity through joint activities with universities and research institutions. We report the data obtained using molecular tools through our close collaboration with the NMP of Botswana to detect active cases of the four *Plasmodium* species that are infectious to humans. We affirm the presence of *P. vivax* and nonfalciparum parasites in Botswana and the changed burden of *P. falciparum* in the country since the 2016 report.

## MATERIALS AND METHODS

### Study sites and population selection.

The study sites were selected with the support of the NMP of the Ministry of Health and Wellness (MOHW) and Ministry of Education of Botswana, as reported previously.^2^ The sites were Okavango, Ngami, Chobe, Tutume, Francistown, Palapye, Serowe, Kweneng West, Kweneng East, and South East. We used a multistage sampling process to select the schools, clinics/health posts, and participants. The Ministry of Basic Education primary school registers and MOHW clinic/health post registries within each district were used to select the schools and clinics involved in the study enrollment.^2^ Briefly, during the first stage, districts with known malaria transmission profiles based on the recommendations of the NMP were selected. During the second stage, towns within the district with variable malaria incidence rates were also selected to ensure heterogeneity within the districts. During the third stage, schools and clinics within each town/village were selected using a two-stage clustering approach based on the population density and adequate cross-sectional representation of the communities to avoid any bias. During the final stage, participants were assigned numbers and enrolled after obtaining informed parental consent and consent from the heads of schools and clinics/health posts. The total number of samples derived for each district/town was in direct proportion to the estimated population density. We enrolled 1,645 children 2 to 13 years of age between December 2015 and February 2017.

### Ethical statement.

The study was approved by the MOHW Ethical Committee of Botswana and the Institutional Review Board of the University of Botswana (permit no. UBR/RES/IRB/BIO/062). All parents/guardians provided informed consent on behalf of all participants. When necessary, assent was also obtained from the child before a sample was collected.

### Sample size.

The sample size was calculated as previously described.^2^

### Selection of subjects.

Community mobilization was performed at Kgotla meetings (community meetings with elders of the community), clinics/health posts, and parent–teacher association meetings before enrollment. Children 5 to 9 years of age were enrolled from primary schools. Children 2 to 4 years of age and 10 to 13 years of age were enrolled from the child welfare clinics. Participants were enrolled only if they had no self-reported clinical symptoms.

Written informed consent for participation in the study was obtained in Setswana or English from parents or guardians for each prospective participant before enrollment. Emphasis was placed on the fact that the participants were free to participate or decline participation without any effect on their medical care.

### Blood sample collection and baseline demographics.

Venous blood samples (1.0–1.5 mL) were collected into EDTA tubes. Hemoglobin concentrations (g/dL) were checked immediately using a Hemocue 201 device (Angelholm, Sweden), and the results were provided to the participants’ caregivers. Participants found to have moderate or severe anemia were referred for further investigation and treatment. Fever was defined as a tympanic temperature ≥ 37.8°C at the time of sample collection; asymptomatic participants were those without fever (tympanic temperature < 37.8°C) and without a history of fever during the preceding 72 hours. Sample collections were timed to include the onset and peak of the malaria transmission season.

The samples were transported in cooler boxes within 3 hours of collection to the district hospital. Blood was separated by centrifugation at 5,000 rpm for 5 minutes into plasma and cell pellets. These were stored at −80°C until they were analyzed.

Demographic variables that were recorded at the time of blood sample collection were age, sex, ethnicity, height, weight, tympanic temperature, place of residence, travel history, and malaria diagnosis during the previous year. Participants who were underweight were referred for further management. Participants who were febrile were also referred to the outpatient department for further management.

### Laboratory analysis.

#### DNA extraction.

Genomic DNA was extracted from the pelleted cells and purified using the Hamilton Star Microlab WorkStation (Hamilton Bonaduz AG, Bonaduz, Switzerland) robotic system. The instructions accompanying the Machery and Nagel (MN) Nucleospin 96 blood kit were followed to achieve the extraction of high-quality DNA. This method uses silica membrane technology for binding DNA after lysis to facilitate purification. The starting blood sample was 200 μL of the packed and thawed erythrocytes, and final DNA elution volume was 120 μL sterile PCR-grade water. Then, 5 μL of the extracted DNA was used for nested PCR and 2.5 μL was used for quantitative PCR (qPCR).

### Molecular detection of *Plasmodium* species

All *Plasmodium* species were detected by modification of the double-nested PCR procedure targeting conserved species-specific regions of the18S small subunit of ribosomal RNA (18S ssRNA) as previously reported.^2,20,21^ For qPCR, the genus-specific primers and probes were those that have been previously described.^21^ The species-specific primers and probes for *P. falciparum*, *P. vivax*, *P. ovale*, and *P. malariae* were also published previously.^22,23^ Samples were screened during the qPCR for positivity and affirmed for species using nested PCR. All nested detection assays were single-plex and performed in a high-throughput 96-well plate (ABI GeneAmp 9700 PCR system; Applied BioSystems, Singapore), and the CFX96 Real-Time PCR detection system (Bio-Rad Laboratories, Pretoria, South Africa) was used for all qPCR assays. The kits used for nested PCR were the Qiagen PCR core kit (Qiagen, Valencia, CA); the KAPA PROBE FAST qPCR Master Mix (2X) (Sigma Inc., Cape Town, South Africa) or the abTES Malaria 5 qPCR II Kit (AITbiotech Pte Ltd, Singapore) were also used for PCR. Species-specific primers and probes were ordered from Eurogentec (Liege, Belgium). Standard controls of *Plasmodium* species 3D7 synchronized cultured parasites were obtained by cell sorting flow cytometry as described previously^24^ (10,000 rings in 50 μL of packed red blood cells). Details of the amplification reactions and cycling parameters for qPCR and nested PCR have been published previously.^25^

### Statistical analysis.

Data were entered in an Excel (Microsoft, Redmond, WA) data sheet. SPSS version 26 (release 2019; IBM Corp., Armonk, NY) was used for analysis. Descriptive statistics and appropriate measures of central tendency were provided for relevant demographic covariates. To describe differences between study sub-populations (e.g., different regions of residence with respect to the presence/absence of *Plasmodium* infection, anemia, and/or age), continuous covariates grouped into categories were compared using binary and multinomial logistic regression. Odds ratios (ORs) were used for logistic regression analyses, and 95% confidence intervals (CIs) were used for point estimates. Anemia was defined as hemoglobin < 11.0 g/dL. Statistical significance for all comparisons was set at *P* < 0.05. Adjustment was not performed for multiple comparisons during this exploratory study.

## RESULTS

### Study population.

**Figure 1.**
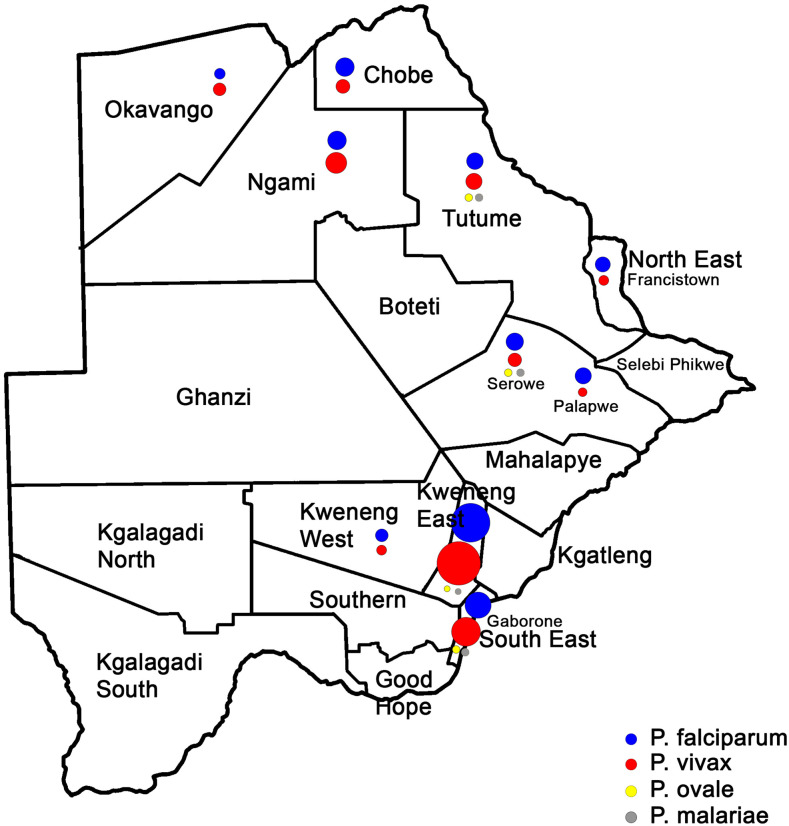
Prevalence of *Plasmodium* species by district.

A total of 1,645 children from 10 districts (Figure 1) were enrolled in the study. Data of 1,614 participants were available for analyses (Table 1). The median age of the participants was 5 years (25^th^ percentile, 3 years; 75^th^ percentile, 8 years). Participants from Tutume (median age, 3 years; 25^th^ percentile, 2 years; 75^th^ percentile, 5 years) were the youngest, followed by those from Kweneng East (median age, 5 years; 25^th^ percentile, 3 years; 75^th^ percentile, 8 years; *P* = 0.005) and Serowe (median age, 4 years; 25^th^ percentile, 3 years; 75^th^ percentile, 7 years; *P* = 0.005). Participants from Ngami (median age, 7 years; 25^th^ percentile, 5 years; 75^th^ percentile, 8 years) and Francistown (median age, 7 years; 25^th^ percentile, 4 years; 75^th^ percentile, 8 years) were older than the other participants (*P* = 0.004). The overall median hemoglobin concentration was 12.1 g/dL; however, anemia was present in the < 20^th^ percentile age group in all districts (*P* < 0.001) (Table 1). Of the patients 2 to 11 years of age (median age, 3.2 years; mean age, 3.95 years), 18.3% had anemia. The risk of anemia decreased with age 5 years or older (OR, 0.26; 95% CI, 0.197–0.34; *P* < 0.001). Anemia was significantly related to the district of residence (*P* < 0.001) (Table 1). Tutume (4.5%) had the most cases of anemia (10^th^ percentile,10.5 g/dL; 25^th^ percentile, 10.5 g/dL; *P* < 0.001), followed by Okavango (*P* = 0.09) and Francistown (*P* = 0.06) (Table 1).). The 10^th^ percentile and 25^th^ percentile hemoglobin concentrations (g/dL), respectively, were 10.2 and 10.7 for Francistown, 10.1 and 11.1 for Chobe, 10.6 and 11.3 for South East, 10.3 and 11.3 for Ngami, 10.5 and 11.4 for Kweneng East, and 10.8 and 11.3 for Serowe. Asymptomatic participants comprised 99.6% of the study population; 0.4% of the symptomatic participants had *P. falciparum* and were clustered mainly in Chobe. There was no difference in the sexes of participants who had *Plasmodium* infections and those who did not.

**Table 1:** Demographics of participants by district of residence

District (n)/Demographics	Age, median (25^th^–75^th^ percentile)	Sex (%)		Hemoglobin, g/dL, median (25^th^–75^th^ percentile)	Total with anemia, %	Total younger than 5 years, %
		F	M			
Okavango (194)	6.0 (4–8)	11.9	12.2	12.3 (11.5–13.1)	1.4	3.8
Ngami (116)	7.0 (5–8)	6.1	8.4	12.0 (11.3–12.7)	1.5	1.6
Chobe (162)	4.5 (4–8)	10.2	9.9	12.0 (11.1–12.7)	2.0	5.0
Tutume (189)	3.0 (2–5)	10.5	13.2	11.4 (10.5–12.3)	4.5	8.3
Francistown (102)	7.0 (4–8)	6.3	6.3	11.9 (10.7–12.8)	1.7	1.6
Palapye (91)	6.0 (3–7)	5.6	5.7	12.5 (11.3–13.2)	0.7	1.9
Serowe (79)	4.0 (4–8)	5.2	4.6	12.3 (11.7–13.6)	0.2	2.5
Kweneng West (148)	6.0 (4–8)	2.7	3.3	12.2 (11.8–12.7)	0.3	1.0
Kweneng East (427)	5.0 (3–8)	28.8	24.1	12.2 (11.4–12.9)	3.9	13.1
South East (206)	4.0 (3–8)	12.7	12.5	12.1 (11.3–12.9)	2.0	6.6

### *Plasmodium* species.

Data regarding *Plasmodium* parasites were available for 1,614 participants (Table 2). All four *Plasmodium* species infectious to humans were observed. The *Plasmodium* parasite infections with *P. falciparum* (12.7%) and *P. vivax* (12.7%) were similar, although the prevalence differed between districts (Table 2). The districts with the highest burden of *Plasmodium* infections were Kweneng East (11.96%), South East (5.82%), Ngami (2.6%), Tutume (2.5%), and Palapye (2.5%); Kweneng West (0.80) and Okavango (0.86%) had the least number of *Plasmodium* infections. All *P. malariae* and *P. ovale* (*curtisi*) infections were present as coinfections with *P. falciparum* or *P. vivax* and clustered in Kweneng East, South East, Tutume, and Palapye. *P. vivax* infections were strongly clustered in Kweneng East (5.6%), South East (2.5%), Ngami (1.3%), and Tutume (0.80%). According to a logistic regression analysis, the three districts with the highest risk of infection with *P. vivax* were Kweneng East (OR, 6.2; 95% CI, 2.94–13.1; *P* < 0.001), South East (OR, 5.6; 95% CI, 2.5–12.3; *P* < 0.001), and Ngami (OR, 5.1; 95% CI, 2.2–12.0; *P* < 0.001). There was also a relatively small cluster at increased risk for infection in Kweneng West (OR, 2.7; 95% CI, 0.8–8.7) and Palapye (OR, 2.5; 95% CI, 0.9–6.8). The risk of infection with *P. vivax* decreased with age in Kweneng East (OR, 0.28; 95% CI, 0.15–0.52; *P* < 0.001), South East (OR, 0.31; 95% CI, 0.16–0.61; *P* = 0.001), and Ngami (OR, 0.35; 95% CI, 0.17–0.74; *P* = 0.006). In contrast, all districts were significantly at risk for infection with *P. falciparum* (*P* < 0.001); however, participants 5 years or older were at decreased risk for infection in all regions except Chobe and Francistown. Interestingly, participants 5 years or older from Okavango were at increased risk for infection (OR, 2.5; 95% CI, −0.94 to 6.7; *P* = 0.066).

**Table 2:** Prevalence of *Plasmodium* species detected per district

District	Samples tested	Type and number of *Plasmodium* species detected									Total from clinics	Total from schools	Parasite prevalence/district
	N	*Pf*	*Pv*	*Pm*	*Po*	*Pv+Pf*	*Pf+Pm*	*Pf+Po*	*Po+Pm*	*Pv+Pm*	N	N	%
Okavango	194	6	8	0	0	0	0	0	0	0	56	138	0.86
Ngami	116	17	21	0	0	4	0	0	0	0	26	90	2.6
Chobe	162	17	10	0	0	3	0	0	0	0	89	73	1.86
Tutume	189	14	13	3	2	1	3	2	2	0	150	39	2.5
Francistown	102	11	5	0	0	2	0	0	0	0	27	75	1.1
Palapye	91	15	9	3	3	1	3	3	3	0	31	60	2.5
Serowe	79	13	4	0	0	0	0	0	0	0	43	36	1.0
Kweneng West	48	8	5	0	0	0	0	0	0	0	17	31	0.80
Kweneng East	427	71	90	3	2	21	2	1	2	1	237	190	11.96
South East	206	33	40	3	3	6	3	3	3	0	117	89	5.82
Total	1614	205	205	12	11	38	11	9	10	1	793	821	
%	100	12.7	12.7	0.74	0.68	2.35	0.68	0.56	0.62	0.06	49.1	50.9	

Pf = *P. falciparum*; Pv = *P. vivax*; Po = *P. ovale*; Pm = *P. malariae*.

## DISCUSSION

The results of our survey demonstrate that the prevalence of asymptomatic *P. falciparum* and *P. vivax* increased significantly during 2016 to 2017, with *P. vivax* accounting for a significant proportion of the asymptomatic *Plasmodium* parasite population in Botswana. Interestingly, this observation was affirmed by the clinical outbreak that led to the conclusion by the WHO, during their assessment, that the malaria elimination agenda of Botswana was off course.^6^ Kweneng East and South East continue to be hotspots for asymptomatic *P. vivax* infections,^2^ which, unfortunately, appear to have spread together with *P. falciparum* into all districts. The presence of significant asymptomatic infections in the population has important implications for strategies and efforts to eliminate malaria. It has been established that ≥ 75% of *Plasmodium* infections are asymptomatic^26^; however, the strategic approach to interrupt *Plasmodium* transmission largely targets symptomatic infections because data from malaria control programs are accrued from passive rather than active case detection.^18^ Asymptomatic infections can be symptomatic during the short term or can remain for long periods^27,28^ before becoming symptomatic.^29^ The caveat is that the majority of asymptomatic infections are also submicroscopic.^17,30,31^ Therefore, only highly sensitive molecular tools can effectively assess their actual extent.^32,33^ It is necessary for malaria elimination programs to change their approach from passive to active case detection with molecular tools,^34,35^ which have the added benefit of being able to detect all *Plasmodium* parasites.^36^

An argument against the detection of asymptomatic infections in NMCPs is based on whether they contribute substantially to transmission. There are transmission hotspots for asymptomatic infections,^37,38^ as have been observed for *P. vivax* and *P. falciparum*, thereby affirming that they do contribute to transmission. It has also been observed that some submicroscopic infections may never progress to symptomatic infections and subtly contribute to transmission.^18^ There are variations in transmission patterns that are geospatially defined in a country, and these variations need to be understood. According to our report in 2016,^2^ Kweneng East, South East, and Tutume were the major hotspots for *P. vivax* infection; however, a new locus of infection for *P. vivax* has emerged in Ngami in the north. Investigations of the Duffy antigen phenotype has been initiated to understand the observed trend.

It is evident that transmission in Okavango has decreased for children younger than 5 years and increased for children 5 years or older. However, it is not clear what the exact reasons are for this observed pattern of infection in this region. Perhaps the persistent efforts by the NMP to mitigate transmission in the district by indoor residual spraying may be a contributory factor. Loci of infections have clearly shifted to the south and southeastern districts, and new strategies are required. The spread of asymptomatic *P. vivax* in the country, including the north, is worrisome because it has significant implications for the elimination agenda because hypnozoites can sustain transmission by serving as infection reservoirs.^37,39^ This also applies to *P. ovale*.^13^ The data regarding Duffy negativity in the population are currently unavailable; however, there is the likelihood that some of the *P. vivax* infections will be Duffy-negative, which will sustain the spread. Therefore, the presence of *P. vivax* and *P. ovale* makes it imperative to implement clear plans regarding the use of primaquine in Botswana. Primaquine has a spectrum of antimalarial activities that leads to the radical cure of hypnozoite stages and sterilization of gametocytes in *P. falciparum*.^40^ We previously reported that the G6PD deficiency in Botswana based on the hemizygous male frequency was 2.30% (95% CI, 1.77–2.83), and that the overall frequency of G6PD-deficient genotype A (hemizygote and homozygote genotypes only) was 1.26% (95% CI, 0.86–1.66); these rates should be considered when deciding to implement the use of primaquine in the country.^41^ *P. vivax* has been labeled as benign, although there is ample evidence to the contrary because of its association with severe malaria.^42^ Perhaps the lack of hyperparasitemia observed with *P. vivax* infections because of the preference for reticulocytes may be a contributing factor.^43^ Recent reports of the presence of *P. vivax* in the bone marrow present the hematopoietic system as a probable hiding place, in addition to the liver, thereby limiting vascular detection by methods other than PCR in nonendemic areas.^43,44^ What we have observed in Botswana can happen in other African countries that have reported the presence of *P. vivax* within the population; therefore, serious attention to this matter is required. An important observation is the level of anemia, which was severer for those younger than 5 years. This observation was related to the district and age, but not the *Plasmodium* infection status; therefore, these anemia cases are more likely to be related to the nutritional status. Nevertheless, we cannot exclude the contribution of existing coinfections. Because anemia can influence infections, and vice versa, particular attention by the NMP is required.^45^

In conclusion, we have shown that *P. vivax* is spreading along with *P. falciparum* in the asymptomatic population in Botswana. Local factors that affect transmission dynamics need to be carefully investigated in Botswana and other African countries. To achieve the elimination of malaria, the NMP in Botswana needs to integrate molecular tools in its strategic planning.
